# Validation of the american quality assessment model and performance improvement to the brazilian transplant*

**DOI:** 10.1590/1518-8345.3249.3252

**Published:** 2020-02-14

**Authors:** Letícia de Fatima Lazarini, Linda Ohler, Janine Schirmer, Bartira de Aguiar Roza

**Affiliations:** 1 Universidade Federal de São Paulo, Escola Paulista de Enfermagem, São Paulo, SP, Brazil.; 2 New York University, Langone Transplant Institute, New York, NY, United States of America.

**Keywords:** Transplantation, Health Management, Quality of Health Care, Health Services Needs and Demand, Validation Studies, Nursing Research, Transplante, Gestão em Saúde, Qualidade da Assistência à Saúde, Necessidades e Demandas de Serviços de Saúde, Estudos de Validação, Pesquisa em Enfermagem, Trasplante, Gestión en Salud, Calidad de la Atención de Salud, Necesidades y Demandas de Servicios de Salud, Estudios de Validación, Investigación en Enfermeira

## Abstract

**Objective:**

To validate the quality assessment and performance improvement instrument of US transplant programs to the Brazilian reality.

**Method:**

Methodological study developed for semantic validation and cultural adaptation of the Quality assessment and Performance Improvement instrument in the following steps: 1) translation; 2) synthesis; 3) back translation; 4) review by expert committee; 5) pretest and 6) content validation. To evaluate the agreement between the five judges, the Kappa coefficient was used and for content validation, the content validation index.

**Results:**

Kappa coefficient showed the agreement of the judges for semantic, idiomatic, cultural and conceptual equivalences. Content validation index values for relevance and item sequence of at least 0.80 for all blocks.

**Conclusion:**

The instrument of Quality Evaluation and Performance Improvement of Transplantation Programs proved to be valid and reliable. This instrument will contribute to the development of quality assurance programs for transplant teams in Brazil.

## Abstract (pt)

**Objetivo:**

validar o instrumento de avaliação da qualidade e melhoria do desempenho dos programas de transplantes dos Estados Unidos à realidade brasileira.

**Método:**

estudo metodológico desenvolvido para a validação semântica e adaptação cultural do instrumento *Quality assessment and Performance Improvement* nas seguintes etapas: 1) tradução; 2) síntese; 3) retrotradução; 4) revisão por comitê de especialistas; 5) pré-teste e 6) validação de conteúdo. Para avaliar as concordâncias entre os cinco juízes, utilizou-se o coeficiente Kappa e para validação de conteúdo, o índice de validação de conteúdo.

**Resultado:**

o coeficiente Kappa mostrou a concordância dos juízes para as equivalências semântica, idiomática, cultural e conceitual. Valores de índice de validação de conteúdo para relevância e sequência de itens de, pelo menos, 0,80 para todos os blocos.

**Conclusão:**

o instrumento de Avaliação da Qualidade e Melhoria do Desempenho dos Programas de Transplantes mostrou-se válido e confiável. Este instrumento irá contribuir para o desenvolvimento de programas de garantia de qualidade para equipes de transplante no Brasil.

## Abstract (es)

**Objetivo:**

validar el instrumento de evaluación de la calidad y mejora del desempeño de los programas de trasplantes de los Estados Unidos a la realidad brasileña.

**Método:**

estudio metodológico desarrollado para validación semántica y adaptación cultural del instrumento *Quality assessment and Performance Improvement* en las siguientes etapas: 1) traducción; 2) síntesis; 3) retrotraducción; 4) revisión por comité de especialistas; 5) pretest y 6) validación de contenido. Para evaluar las concordancias entre los cinco jueces, se utilizó el coeficiente Kappa y para la validación de contenido, el índice de validación de contenido.

**Resultado:**

el coeficiente Kappa mostró la concordancia de los jueces para las equivalencias semántica, idiomática, cultural y conceptual. Los valores de índice de validación de contenido para relevancia y secuencia de ítems, de por lo menos, 0,80 - para todos los bloques.

**Conclusión:**

el instrumento de Evaluación de la Calidad y Mejora del Desempeño de los Programas de Trasplantes se mostró válido y confiable. Este instrumento podrá contribuir para el desarrollo de programas de garantía de calidad para los equipos de trasplante en Brasil.

## Introduction

The World Health Organization (WHO) in recent years has developed and encouraged the development of quality programs in health institutions with the principle that each person has the right to receive the best possible care, equally^(^ 1 ^-^ 2 ^)^. Thus, WHO has taken a leading position in facilitating and scaling up different approaches to quality within the health system. Additionally, in most countries, quality is considered a strategic component, regardless of the level of economic development or the type of health system^(^ 1 ^-^ 2 ^)^. Therefore, we must overcome the conception that considers the discourse of quality as the prerogative of rich countries with an advanced health system. Thus, quality in health care today is part of the national and international agenda and is present in the debates on health system reform.

Despite this fact, Brazil does not have a quality program or policy for the process of organ and tissue donation and transplantation that can determine the causes of losses due to underreporting, maintenance and family refusal, as a result of the care process, and indicators. pre and post-transplant outcome as well as patient survival^(^ 3 ^)^. Even so, in 2017, Brazil performed 5,929 kidney transplants and 2,109 liver transplants^(^ 4 ^)^, while the United States performed 19,849 kidney transplants and 8,082 liver transplants in the same period, according to the United Network for Organ Sharing (UNOS). 

A study published in 2015 on the validation of instruments of the National Transplant Organization (NTO) Quality Management Model^(^ 5 ^)^ catalyzed the implementation of the quality model in the state of São Paulo as a management policy. Such a model aims to prevent donor loss in state hospitals by completing an electronic report. Thus, there is tracking of organ donation teams with the possibility of improvement. In 2017, the State of Santa Catarina achieved the highest effective donation rate in the country, with 40.8 parts per million population (pmp), followed by the state of Paraná with 38.0 pmp^(^ 4 ^)^. Despite national advances in organ donation and transplantation, investments in transplantation system research and auditing are needed to improve the quality of organ donation and transplantation processes, increase patient quality of life, and reduce costs and increased patient safety^(^ 6 ^-^ 7 ^)^.

Organ and tissue transplantation, funded by the Unified Health System (UHS), which has no budget ceiling constraint, is not subject to regular evaluation either, and when evaluated, quality indicators based only on patient and graft survival are used, leaving aside important assessments such as cost-effectiveness. Decree No. 9,175 of the Civil House of October 2017, for example, regulates, in its article 8, that State Transplant Centers (STC) must “define, together with the central organ of the National Transplant System (NTS)”, parameters and quality indicators for the evaluation of transplantation services, histocompatibility laboratories, tissue banks and organisms that are part of the search and donation network for organs, tissues, cells and parts of the human body”. 

Therefore, in order to improve Brazilian transplantation policies, the Ministry of Health and the professionals involved in donation and transplantation processes should rethink the quality indicators used by transplantation teams aiming at indicators that demonstrate the condition of patients and the use of assistance processes, allowing future integral cost-benefit evaluations, ethical process responsibility and transparency of public and social investment. International collaborations are beneficial for this purpose, because they have models of care and evaluation that have already been used in a given population. The adaptation of such models is a common practice that aims to use the scientific evidence generated for the Brazilian reality, avoiding the duplication of unnecessary studies and reducing research costs. One model that could be useful for the Brazilian reality is the quality assessment and performance improvement instrument of transplantation programs used in the United States of America (the American model for quality assessment and improvement (8)). Adapting it to the Brazilian reality would contribute to improving the performance of the Brazilian transplantation system. The Quality Assessment and Performance Improvement (QAPI) instrument is used to determine the compliance of transplantation programs with current legislation and technical regulations in the area of transplantation. This instrument is designed to verify that transplantation teams understand the legislation and technical regulations to align expectations with national agencies to transplantation teams and to provide researchers with quality assessment tools^(^ 8 ^)^.

Data selection for outcome analysis and each team’s process for quality assessment and performance improvement are visible in the QAPI instrument. Each team can use the most effective and best suited method, as there is no obligation to use specific tools for each item analyzed, as the focus is the result^(^ 8 ^)^. Thus, this study aims to validate the quality assessment and performance improvement instrument of United States transplantation programs to the Brazilian reality.

## Method

This is a methodological study for semantic validity and cultural adaptation of a measuring instrument through the process of translation, adaptation and content validation of the questionnaire. The instrument used for translation was the Quality Assessment and Performance Improvement (QAPI)^(^ 8 ^)^. This instrument has 64 open questions about the indicators used by transplantation teams divided into the following parts: Team identification; 1 - Program policies and procedures; 2 - Program evaluation and monitoring; 3 - Review of indicators; 4 - Actions/Activities of performance improvement and resolution of nonconformities, complaints and side effects; 5 - Transplantation and Final Program Adverse Event Policies/Procedures and Analysis and 6 - Quality Assessment and Performance Improvement Decision Making.

The study was submitted and approved by the Research Ethics Committee of the Federal University of São Paulo under the opinion 1571621 CEP 0570/2016. In consultation with the Center for Clinical Standards and Quality Survey & Certification Group, formal authorization to use the instrument was waived because it is a public domain instrument.

For the validation of this instrument, the proposal by Beaton et al., which provides the following methodological steps, was used^(^ 9 ^)^: translation; synthesis; back translation; expert committee review; pretesting and content validation. 

The translation was performed from the original instrument by two professionals with mastery of the Brazilian Portuguese language, one professional in translation and one health professional^(^ 9 ^)^. From both versions, the synthesis was generated by the researcher and judges. 

After the synthesis, the Portuguese-language instrument was translated to the source language by two translators, one American health professional and one native speaker of the English language, and thus the new English version was compared to the original version (back-translation)^(^ 9 ^)^. After the instrument had been translated, synthesized and back-translated, it was sent by e-mail and evaluated by a committee of experts composed of five nurses, national references in the area of transplantation and in the area of health quality evaluation, for validation of content. To be considered national references in the area of transplantation, professionals with more than two years of experience in organ transplant management, with academic experience and validation of research instruments and mastery of the American English language were selected. 

A first evaluation was performed by the experts about the content of the questionnaire descriptively and from their considerations. Secondly, content validation was performed using a Likert scale,^(^ 10 ^)^ graded from “one” to “five”, between totally disagree to totally agree, respectively. The following items were evaluated: relevance to the proposed objective; clarity in the wording of the items; accuracy, i.e. the accuracy of each question; objectivity as to the assertiveness of each item. The sequence of items in which each question appears in the questionnaire was also evaluated, in addition to the semantic equivalence criteria, ie the meaning of words and the idiomatic equivalence in the use of colloquial idioms, cultural equivalence in terms of colloquially used expressions typical of the culture of the place and, finally, the conceptual equivalence, that is, as the concept required in the questionnaire^(^ 11 ^-^ 12 ^)^. 

To perform content validation, the Content Validity Index (CVI) was used, which evaluates the proportion of judge agreement^(^ 12 ^)^ about each item and about each part of the instrument. The CVI of each part of the instrument (consisting of at least two items and a maximum of 26 items) was obtained via the mean CVI values of each item. Similarly, the total CVI corresponds to the average CVI of each part of the equivalences represented in the instrument. For this research, answers above “four” were considered relevant, with variability from “one” to “five” and items above 0.8^(^ 12 ^)^.

After the evaluations of the instrument made by the five judges, the final version for the pilot test was generated, adopting each consideration made, and the statistical analysis of the data was performed by calculating the Kappa coefficient^(^ 13 ^)^ for multiple judges. 

The questionnaire was subjected to a pilot test^(^ 11 ^)^ with ten specialist health professionals and transplantation references from different states of Brazil (Brasilia, Curitiba, Belem, Campo Grande, Botucatu, Sorocaba and São Paulo). This number of participants is due to the complexity of the area and the logistical difficulty, considering that the pilot test was applied to people with proven experience in the area and decisive role in donation and transplantation in the country. The choice of the professionals was based, besides the experience of transplantation work in the different Brazilian regions, on the number of transplants performed by the institutions in which the professionals worked. 

The ten selected professionals responded to the QAPI instrument and, at the end of the pilot test, based on all the considerations made by the experts, the final version of the Instrument for Quality Evaluation and Performance Improvement of Transplantation Programs for Brazil was obtained.

## Results

During the translation and synthesis phase of the questionnaire, no major changes were made, as the original layout of the questionnaire was respected. In the synthesis phase, one favored the option that fit culturally to the Brazilian standards of transplantation teams, which presented a clearer language and adapted to the Brazilian transplantation teams.

The profile of the judges selected for this research had a mean age of 35 years, with approximate experience in the transplant process of five years, and 20% of the judges worked directly in the transplant process and 40% also worked in teaching and search. Of these professionals, 60% had a master’s or doctorate degree and 40% had already participated in questionnaires/instrument/scales validation work.

During the judges’ evaluation, the use of the word “only” to identify the types of transplantation performed by transplantation teams generated doubt and unanimously decided to remove this word, without prejudice to the question. The panel of judges also removed “heart/lung” as the only double transplant option in the questionnaire, and such options already appeared individually in other items. The word “lawyer” was replaced by “supporter”, a term already used in Brazil by transplant teams.

The adaptation of the QAPI instrument presented 0.97 of semantic and idiomatic equivalence and 0.99 of cultural and conceptual equivalence corresponding to Identification; Program policies and procedures; Program evaluation and monitoring; Review of indicators; Actions/Activities of performance improvement and resolution of nonconformities, complaints and side effects; Transplantation and Final Program Adverse Event Policies/Procedures and Analysis.

No item related to equivalence was below 80%, however, the considerations made by the judges regarding the adequacy of the questionnaire were respected. Although the CVI reached over 80% for equivalences, the judges’ considerations were about the layout of the tables and spaces to answer the notes.

For the blocks in which it was possible to calculate the Kappa coefficient, the agreement showed that there was no variation in the judges’ score, as shown in table 1. However, the agreement observed between the judges regarding the adequacy of semantic, idiomatic, cultural equivalence and Conceptual Identification Blocks, Program Policies and Procedures, Program and final evaluation and monitoring, as well as cultural and conceptual adequacy of the Program Policies and Procedures, Program Evaluation and Monitoring, and Review of Indicators were 100.0%

**Table 1:** Kappa coefficient for multiple judges according to equivalence. Sao Paulo, SP, Brazil, 2017.

Item	Equivalence				Items
	Semantics	Idiomatic	Cultural	Conceptual	
Identification	-*	-*	-*	-*	6
Policies and procedures	-*	-*	-*	-*	14
Evaluation and monitoring	-*	-*	-*	-*	2
Indicator Review	-0,091	-0,091	-*	-*	12
Performance Improvement Actions / Activities	-0,25	-0,25	-0,25	-0,25	2
Adverse Event Policies / Procedures and Analysis	-0,008	-0,008	-0,008	-0,008	26
Final	-*	-*	-*	-*	2
Total	-0,026	-0,026	-0,01	-0,01	64

* Unable to calculate Kappa coefficient - all pointed out to be an important item

For items considered equivalent, according to the CVI, with significant equivalence equal to or above 0.8 of approval, no change was required, but for items below 0.8 of approval, change was required according to the considerations made by the judges. Thus, the tables of transplantation process indicators, transplantation results, live donor process and live donor outcome, transplantation program actions and activities, and noncompliance resolution were modified, adding more space to respond and the terms name of the Institution’s Transplant Program physician and nurse coordinator, living donor supporter and notification to the National Health Surveillance Agency (ANVISA). 

The CVI values for relevance, accuracy, clarity, objectivity and sequence of items were 0.97, 0.85, 0.82, 0.86, 0.93, respectively, for all blocks. Total CVI was 0.89 for all items, demonstrating a questionnaire item validity index of at least 0.80 in each block. 

A moderate agreement (k=0.422; p<0.001) for the objectivity of part one is seen in table 2, as well as the objectivity in parts two and five and a good sequence of topics for identification, part two and part five.

**Table 2:** Kappa coefficient for multiple judges according to relevance, accuracy, clarity, objectivity and sequence of items per block. Sao Paulo, SP, Brazil, 2017.

Item	Relevance	Precision	Clarity	Objectivity	Sequence of Items	Itens
Identification	-*	-0,01	-0,108	-0,08	-*	6
Policies and procedures	-0,015	0,109	0,242^†^	0,422^‡^	0,358^‡^	14
Evaluation and monitoring	-0,25	0,063	0,063	-*	-*	2
Indicator Review	-*	0,008	0,025	-0,071	-0,154	12
Performance Improvement Actions / Activities	-0,25	-0,2	-0,2	-0,042	-0,25	2
Adverse Event Policies / Procedures and Analysis	-*	-*	-*	-*	-*	26
Final	-0,25	-0,25	-0,25	-0,25	-0,25	2
Total	-0,011	0,165	0,210‡	0,249‡	0,146†	64

* It was not possible to calculate the Kappa coefficient - all pointed out to be an important item.

† p value <0.010.

‡ p value <0.001

Regarding the considerations of the ten professionals who completed the Quality assessment and Performance Improvement (QAPI) instrument, the size of the questionnaire was mentioned, as 30% classified it as too large to be completed monthly and, as a justification, attributed the large number of assignments that the nurse coordinator of each team needs to develop.

It was also observed that 20% of professionals rated the instrument as difficult to be answered in the pilot test, but the objective of the instrument is to provide indicators for transplantation programs to perform quality management to improve transplantation processes. In this phase, it was also noticed the difficulty of the teams in grouping the data available to them. Some teams were in the implementation phase of indicators and others did not have them. Professionals from the city of São Paulo and inland were easier to answer the instrument without question. However, 20% of professionals did not answer the questions related to adverse events, as the program did not have the data.

## Discussion

Quality management is currently being discussed in health care as a key to transplant outcomes and is beginning to integrate research and add value to patient care. It is expected to promote quality care in the routine of transplant centers with practices defined by the quality management model^(^ 14 ^-^ 15 ^)^. American examples are considered effective and favor management for transplant teams^(^ 16 ^)^.

This study had qualified professionals to collaborate with the validation of the QAPI instrument, and 80% of the professionals felt that the instrument is of great relevance for transplantation in Brazil. As well as other international instrument validation studies^(^ 17 ^-^ 18 ^)^, This study had to adapt the instrument’s language to the Brazilian reality in donation and transplantation. In the United States, it is common for patients to have “advocates” who support the living donor patient on their donation path^(^ 19 ^)^. In Brazil, although there is no such nomenclature, living donors rely on health network supporters, such as social workers and psychologists, during their decision to donate. Then the judges suggested changing the term from “lawyer” to “supporter”. Although such modifications were necessary, the questionnaire generally showed high agreement when compared to other similar studies^(^ 18 ^,^ 20 ^)^ on semantic, idiomatic, cultural, and conceptual equivalence adequacy of the identification blocks, Program Policies and Procedures, Program Evaluation and Monitoring, and Cultural and Conceptual Adequacy. 

In the pre-test of the study, there was a discrepancy between the State of São Paulo and the other states regarding the creation of indicators and notification of adverse events, which characterizes a critical point for the education and qualification of transplantation teams. Studies in this sense tend to guide the nurse professional to notify and organize actions to prevent adverse events^(^ 21 ^-^ 22 ^)^. 

The indicators are intended to measure qualitative and / or quantitative aspects related to the environment, structure, processes and results. The indicator by itself does not represent a direct measure of quality, but indicates attention to specific outcome issues within a healthcare organization^(^ 23 ^)^. The management of the organ donation and transplantation process in the country is defined by Ordinances No. 1.262, which approved the technical regulation to establish the attributions, duties and indicators of efficiency and the potential of organ and tissue donation related to the In-Hospital Commissions of Donation of Organs and Tissues for Transplant (CIHDOTT), and Ordinance No. 2,600, 2009, which approved the technical regulation of the NTS. However, the failure to present results, through indicators, by transplantation teams in Brazil, public managers, to issue and maintain their records, may explain the low adherence to quality programs in this health area.

The recent publication of Decree No. 9.175 of 18 October 2017 may change the history of donation and transplantation in the country, by requiring a quality management process, which will need to be incorporated by transplantation centers, in which instruments for the evaluation of quality will be needed to compile information on care processes with the obligation of transparency to the society that finances them. 

This study leaves as a contribution to the advancement of scientific knowledge, a validated instrument that aims to facilitate, to health professionals, the data management of their transplantation teams, in a uniform and effective way, benefiting patients and health professionals. It will also make it possible to give society visibility with the dissemination of results of a care process guaranteed by UHS.

As a limitation of the study, the instrument presents open questions without a score, which made the statistical analysis difficult. Another difficulty, due to the organization of the transplantation teams, was that not all regions of the country participated in the study in the pilot study phase because they did not use quality evaluation indicators during the study period.

## Conclusion

This study successfully completed the Portuguese language validation of the Quality assessment and Performance Improvement (QAPI) instrument. The instrument proved to be effective for the purpose of compiling the transplantation program data to the Brazilian reality. Future efforts to apply such an instrument to the Brazilian reality will have the potential regulatory effect of transplantation on quality of care, including work in the areas of risk adjustment, outcome reporting, access to care, and health economics.
